# Lactonase Activity and Lipoprotein-Phospholipase A_2_ as Possible Novel Serum Biomarkers for the Differential Diagnosis of Autism Spectrum Disorders and Rett Syndrome: Results from a Pilot Study

**DOI:** 10.1155/2017/5694058

**Published:** 2017-11-28

**Authors:** Joussef Hayek, Carlo Cervellati, Ilaria Crivellari, Alessandra Pecorelli, Giuseppe Valacchi

**Affiliations:** ^1^Child Neuropsychiatry Unit, University General Hospital, Azienda Ospedaliera Universitaria Senese, Viale M. Bracci 16, 53100 Siena, Italy; ^2^Department of Biomedical and Specialist Surgical Sciences, University of Ferrara, Via Luigi Borsari 46, 44121 Ferrara, Italy; ^3^Department of Life Sciences and Biotechnology, University of Ferrara, Via Luigi Borsari 46, 44121 Ferrara, Italy; ^4^Plants for Human Health Institute, Animal Science Department, NC Research Campus, NC State University, 600 Laureate Way, Kannapolis, NC 28081, USA

## Abstract

Rett syndrome (RTT) and autism spectrum disorders (ASDs) are not merely expression of brain dysfunction but also reflect the perturbation of physiological/metabolic homeostasis. Accordingly, both disorders appear to be associated with increased vulnerability to toxicants produced by redox imbalance, inflammation, and pollution, and impairment of systemic-detoxifying agents could play a role in the exacerbation of these detrimental processes. To check this hypothesis, the activities of two mechanistically related blood-based enzymes, paraoxonase-1 (arylesterase, paraoxonase, and lactonase), and lipoprotein-associated phospholipase A_2_ (Lp-PLA_2_) were measured in the serum of 79 ASD and 95 RTT patients, and 77 controls. Lactonase and Lp-PLA_2_ showed a similar trend characterized by significantly lower levels of both activities in ASD compared to controls and RTT (*p* < 0.001 for all pairwise comparisons). Noteworthy, receiving operator curve (ROC) analysis revealed that lactonase and, mostly, Lp-PLA_2_ were able to discriminate between ASD and controls (lactonase: area under curve, AUC = 0.660; Lp-PLA_2_, AUC = 0.780), and, considering only females, between ASD and RTT (lactonase, AUC = 0.714; Lp-PLA_2_, AUC = 0.881). These results suggest that lactonase and, especially, Lp-PLA_2_ activities might represent novel candidate biomarkers for ASD.

## 1. Introduction

Rett syndrome (RTT), the second most prevalent cause of severe mental retardation in female gender (frequency among females: 1 : 10,000 to 1 : 15,000), is a progressive neurodevelopmental disorder [1]. The classic form of RTT (affecting the 95% of total cases) is caused by specific mutations in the X-linked gene encoding the methyl-CpG-binding protein 2 (*MECP2*) [2]. Patients affected by RTT typically exhibit various neuropsychiatric features after 6–18 months of apparently normal neurodevelopment, including pervasive growth failure, progressive cognitive impairment (with loss of previously acquired speech), and replacement of purposeful use of the hands with incessant stereotypies (hand wash like) [3].

Autism spectrum disorder (ASD) prevalence has dramatically increased in the last decades, mostly due to the expansion of diagnostic criteria. The most recent data have shown an overall estimate of 7.6 per 1000 children worldwide (14.7 in the US) [4]. This heterogeneous group of neurodevelopmental disorder is now regarded as the result of a complex and still not fully understood interaction between a genetic background and environmental factors. ASDs occur predominantly in males (four times more) with a clinical onset usually within the 2nd year of life and characterized mainly by severe impairment in reciprocal social interactions and communication skills and the presence of restricted stereotypical behaviors. Classical autism is the predominant ASD phenotype, also including Asperger syndrome, pervasive developmental disorder not otherwise specified (PDD-NOS), and childhood disintegrative disorder [5]. Of note, RTT has been recently removed from the ASD (diagnostic and statistical manual of mental disorders V-DSMV).

ASDs and RTT have several common characteristics, and the respective clinical manifestations are almost undistinguishable during the regression period of RTT, which displays transient autistic features, lasting from weeks to months. Moreover, as highlighted by MRI-based studies, brain morphology characterizing RTT and ASDs shares several features such as smaller neuron size and simplified, reduced dendritic spines, and branches without neuronal loss during clinical progression of the disease [3, 6]. Consistent with this line of reasoning, it is now well accepted that cognitive/behavioral diseases, including RTT and ASDs, are not merely expression of central nervous system (CNS) dysfunction but might also reflect the disruption of physiological/metabolic homeostasis [7–9]. Indeed, there is plenty of evidence suggesting that inflammation, impaired detoxification, and altered redox homeostasis [9–11] could play a crucial pathogenic role in both RTT and ASDs; therefore, finding a possible common denominator, or discriminatory factor, would be of extreme interest even for early diagnosis.

Paraoxonase-1 (PON-1) is a pleiotropic enzyme associated with high-density lipoprotein (HDL) that contributes to the systemic protection against toxic agents deriving from oxidative stress (OxS), exacerbating inflammatory response, dyslipidemia, and pollution exposure [12, 13]. The ability to counter the effects of a wide range of noxious agents appears to be linked to the broad substrate specificity that characterizes PON-1 [14]. This enzyme elicits three distinct hydrolytic activities: (1) paraoxonase, towards toxic organophosphates such as paraoxon, the toxic oxon metabolite of parathion, an insecticide; (2) arylesterase, towards nonphosphorous aryl esters, such as phenyl acetate; and (3) lactonase, towards lactones [14]. While paraoxonase and arylesterase, being toward man-made substrates, are classically referred as too promiscuous activities, lactonase is now widely suggested to be the primary activity of PON-1 [15]. The physiological substrate of PON-1 is still uncertain, but there is some evidence suggesting the endogen lipophilic lactones such as those resulting from fatty acid oxidation from phospholipid (PLP), cholesteryl ester, and triglyceride hydroperoxides or from homocysteine metabolic pathway [14, 16]. However, it is fair to underscore that neither the catalytic mechanism(s) nor the physiological (antioxidant) effect of this (lipo-)lactonase activity is still completely understood [15].

Lipoprotein-associated phospholipase A_2_ (Lp-PLA_2_) is another enzyme circulating in complex with HDL and, mostly, low-density lipoprotein (LDL) that plays multiple roles in redox and inflammatory processes [17]. Nevertheless, as also highlighted for PON-1, the mechanism of action and the biological role of Lp-PLA_2_ are still not clear. This enzyme catalyzes the hydrolysis of acetyl group at the sn-2 position of platelet-activating factor (PAF), that is, its endogen natural substrate, thereby inactivating this proinflammatory PLP [17]. In vitro evidence suggests that Lp-PLA_2_ is also able to degrade other phospholipids (PLPs), resembling PAF structure, containing oxidized fatty acyl groups, to form lysophospholipids and acetate and oxidized fatty acid [18]. Despite the abundant studies on the topic, it is still not clear whether high levels of Lp-PLA_2_ are beneficial or detrimental for human health. Indeed, from one hand, this enzyme is highly expressed in nascent atherosclerotic plaques and is involved in multiple stages of atherosclerosis, and this explains the positive association between Lp-PLA_2_ activity and increased risk of coronary heart disease [18–20], while, from the other side, parallel studies have clearly shown that overexpression of this enzyme reduces atherosclerosis in mice [21] and in rabbits [22].

There is a wealth of evidence showing that PON-1 and Lp-PLA_2_ levels are altered in diseases apparently characterized by the detrimental crosstalk between altered redox homeostasis and chronic inflammation [12, 19, 23–27]. By contrast, we found only a handful of studies on PON-1 in ASDs patients, reporting lower levels of arylesterase (paraoxonase unchanged or lower, lactonase not measured) in affected individuals compared to controls [28–31]. To the best of our knowledge, there are no published data on PON-1 and Lp-PLA_2_ activities in RTT or ASD. To bridge this gap of knowledge, in this study, we sought to determine whether alteration in the activities of these two lipoprotein-associated enzymes might be selectively altered in RTT and ASDs.

## 2. Materials and Methods

### 2.1. Subjects

The subjects enrolled in the study included *n* = 94 female patients with clinical diagnosis of RTT (all with MECP2 mutation), *n* = 76 patients with ASD, and *n* = 78 healthy controls (age and gender prevalence across the groups are presented in Table 1).

This research protocol was carried out accordingly to the Declaration of Helsinki (World Medical Association, http://www.wma.net), and the European Guidelines for Good Clinical Practice (European Medicines Agency, http://www.ema.europa.eu). The study did not modify the routine implemented for the diagnosis of RTT or ASD nor conditioned any decision about the treatments of the enrolled individuals, and it was approved by the local institutional review board. Written informed consent was obtained from each patient during the first office visit at baseline before the possible inclusion in the study.

Blood sampling from controls was performed using routine health checks, sports checkups, or through blood donations. All the patients (ASD and RTT) were consecutively admitted to the Child Neuropsychiatry Unit of the University Hospital of Siena (Azienda Ospedaliera Universitaria Senese), and blood samplings were performed during periodic clinical checkups. RTT diagnosis and inclusion/exclusion criteria were based on the recently revised RTT nomenclature consensus [32]. Of note, 87 of 95 (91%) girls with RTT were unable to speak.

The autistic patients were diagnosed by DSM-5 and evaluated using Autism Diagnostic Observation Schedule (ADOS) and Autism Behavior Checklist (ABC). ASD patients with diagnosed X-fragile or tuberous sclerosis, with perinatal adverse events and/or brain abnormalities on magnetic resonance imaging (MRI) were excluded from the present study. ASD patients under medication or pharmacological treatment at the time of blood withdrawal were not included in the study.

### 2.2. Biochemical Analysis

Fasting venous blood was collected in the morning, and all manipulations were carried out within 2 hrs; then, the sera were aliquoted and stored at −80°C until analysis.

Serum lactonase, paraoxonase, arylesterase activities of PON-1, and Lp-PLA_2_ were measured by UV–VIS spectrophotometric assays in a 96-well plate by using a Tecan Infinite M200 microplate reader (Tecan Group Ltd., Switzerland).

Paraoxonase activity assay was performed by measuring the rate of hydrolysis of paraoxon by monitoring the increase of absorbance at 410 nm after adding 10 *μ*L of serum. The assay reagent contained 1.5 mmol/L paraoxon (Sigma-Aldrich) and 1 mmol/L CaCl_2_ in 10 mmol/L glycine buffer (pH = 8) [24, 33]. A molar extinction coefficient of 17,000 M^−1^ cm^−1^ was used for the calculation of enzyme activity, expressed in units per liter. One unit of paraoxonase activity is defined as 1 *μ*mol of 4-nitrophenol formed per minute under the given conditions.

Arylesterase activity was measured by assessing the rate of hydrolysis of phenylacetate by monitoring the increase of absorbance at 270 nm, after adding 10 *μ*L of serum (diluted 24 times). The reaction mixture was composed by 1 mmole/L phenylacetate (Sigma-Aldrich) and 0.9 mmol/L CaCl_2_ dissolved in 9 mmol/LTris-HCl, pH = 8 [23, 33]. A molar extinction coefficient of 1310 M^−1^ cm^−1^ was used for the enzyme activity calculation, expressed in kilo unit per liter. One unit of arylesterase activity accounts for 1 *μ*mol of phenol produced in a minute under the conditions of the assay.

PON-1 lactonase activity was measured by using gamma-thiobutyrolactone (TBL, Sigma-Aldrich) as substrate, and Ellman's procedure was used to spectrophotometrically monitor (412 nm) the accumulation of free sulfhydryl groups via coupling with 5,5-dithiobis(2-nitrobenzoic acid) (DTNB), as described elsewhere [25]. The reaction was started by adding 10 *μ*L of sample to the reaction mixture containing buffer (50 mmol/L Tris, 1 mM CaCl2, 50 mmol NaCl, pH = 8), 0.5 mmol/L DTNB, and 10.5 mmol/L TBL in each well. A molar extinction coefficient of 13,600 M^−1^ cm^−1^ was used for the enzyme activity calculation, expressed in unit per liter.

Lp-PLA_2_ was assessed by using 2-thio PAF as substrate, which is hydrolyzed by the enzyme in sn-2 position. The consequent formation of free thiols was detected by Ellman's procedure. The reaction was started by adding 10 *μ*L of sample to the reaction mixture containing buffer (100 mM Tris, 0.1 mmol/L ethylene glycol-bis(2-aminoethylether)-N,N,N′,N′-tetraacetic acid (EGTA), pH = 7.2), 0.5 mmol/L DTNB, and 0.2 mmol/L 2-thio PAF (Cayman Chemical, Ann Arbor, Michigan US) in each well. A molar extinction coefficient of 13,600 M^−1^ cm^−1^ was used for the enzyme activity calculation, expressed in unit per liter.

### 2.3. Statistical Analysis

Means of the variables examined were compared by using analysis of variance (ANOVA plus Sidak post hoc test for pairwise comparisons), after checking for normal distribution by Kolmogorov-Smirnov test. Since the distribution of paraoxonase and Lp-PLA_2_ was skewed, the values were log transformed in order to approximate a normal distribution before being analyzed by parametric analyses, such as ANOVA. Prevalence of categorical variables was compared by the *χ*^2^ test. Analysis of covariance (ANCOVA plus Sidak post hoc test) was performed to check if the differences revealed by univariate analysis were influenced by gender. Receiver operating characteristic (ROC) analysis was performed to determine the ability of parameters examined to discriminate between RTT/ASD and control and between RTT and ASD. A *p* < 0.05 was considered statistically significant.

## 3. Results

### 3.1. Comparison of Serum PON-1-Related and Serum Lp-PLA_2_ Activities between Controls, RTT, and ASD

**Figure 1:**
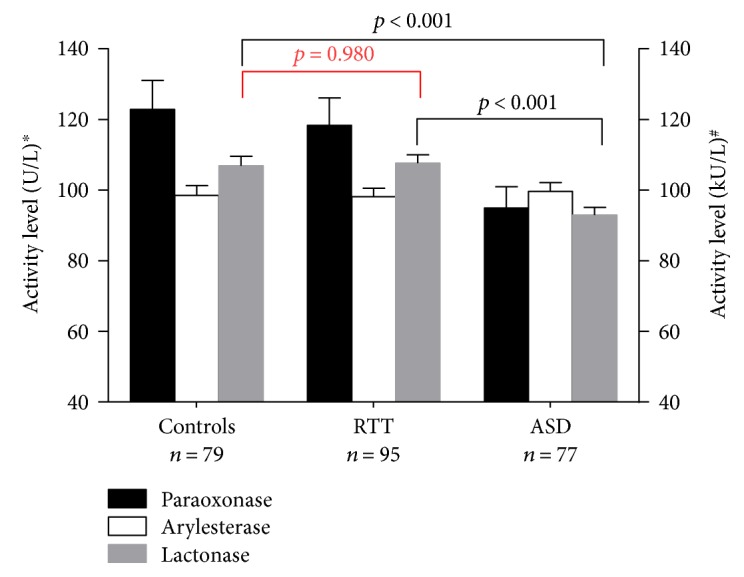
Serum paraoxonase, arylesterase, and lactonase activities in control, RTT, and ASD patients. The graph with bars ± SD shows PON-1 activities in the three groups. ^∗^Lactonase and paraoxonase activities are expressed as U/L. ^#^Arylesterase activity is expressed as kU/L.

PON-1-related activities had a comparable trend of lactonase (ANOVA: *p* < 0.001) and paraoxonase (Kruskal-Wallis: *p* = 0.04) activities across the sample groups (Figure 1). The pairwise differences were significant only for lactonase, with levels decreased by approximately 14% in ASD and RTT (*p* < 0.001 for both pairwise comparisons) as compared to controls.

**Figure 2:**
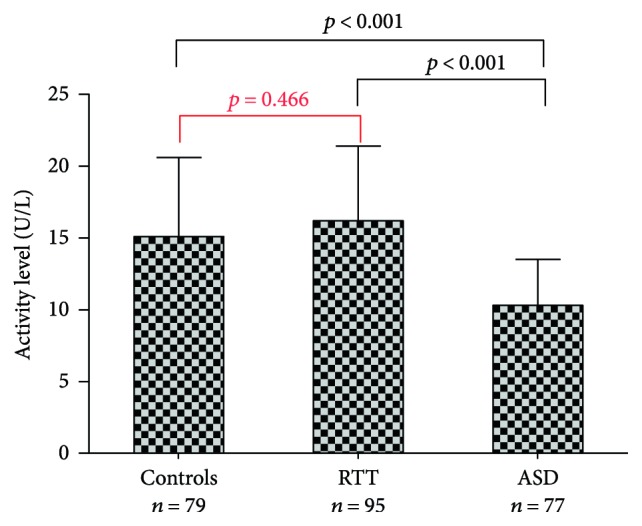
Serum Lp-PLA_2_ activity in control, RTT, and ASD patients. Lp-PLA_2_ activity is reported in U/L +/− SD.

Similar to paraoxonase and lactonase activities, serum Lp-PLA_2_ reached the lowest level in the ASD patients (Figure 2). To be noted, the decrease of Lp-PLA_2_ was more evident (−32% and −37%, with respect to controls and RTT patients) to that observed for the PON-1 activities. Of note, we did not find any significant association between PON-1 activities/Lp-PLA_2_ and ADOS scores among ASD patients.

### 3.2. Study of the Possible Effects of Gender on Statistical Outcomes

Owing the evident differences in the female/male distribution among the groups examined (Table 1), we checked whether the changes in Lp-PLA_2_ and lactonase activities revealed at univariate analysis were influenced by gender. These analyses (data not shown) showed that, after adjustment for gender, lactonase was still significantly lower in ASD compared to controls (*p* < 0.05) and RTT (*p* < 0.001), with the latter difference becoming even more dramatic. Alike, Lp-PLA_2_ remained significantly lower in ASD than in the other two sample groups (*p* < 0.001 for both comparisons). Of note, the significant levels of the above-described differences in lactonase and Lp-PLA_2_ were not affected by age.

### 3.3. Comparison of Serum Lactonase and Serum Lp-PLA_2_ Activities among Controls, RTT, and ASD in Female Subsample

**Figure 3:**
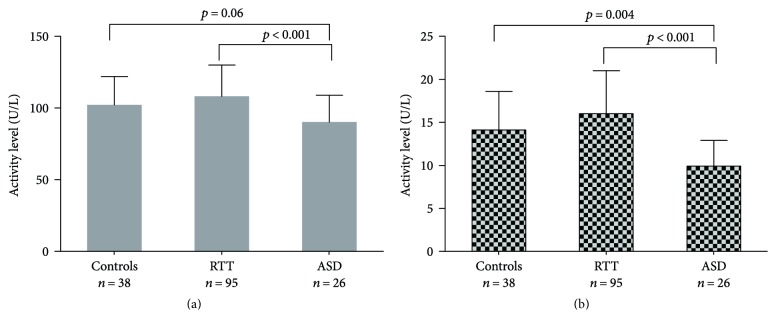
Serum lactonase (a) and Lp-PLA_2_ (b) activities in control, RTT, and ASD female patients (+/− SD).

Since RTT group was completely composed by girls and ANOVA was not the most proper approach to check potential differences between RTT and the other two, we compared lactonase and Lp-PLA_2_ levels only considering the females present in the ASD and control sample (*n* = 157). As displayed in Figure 3, lactonase activities remained significantly lower in ASD compared to RTT (*p* = 0.001), while the gap between the former and controls was reduced (when compared to total sample) and almost reached a significant threshold (*p* = 0.06). The proportions were preserved also for Lp-PLA_2_, which resulted markedly lower among ASD with respect to both controls and RTT (*p* < 0.001 for both comparisons).

### 3.4. Lactonase and Lp-PLA_2_ as Possible Diagnostic Biomarkers: Outcomes of ROC Analyses

**Figure 4:**
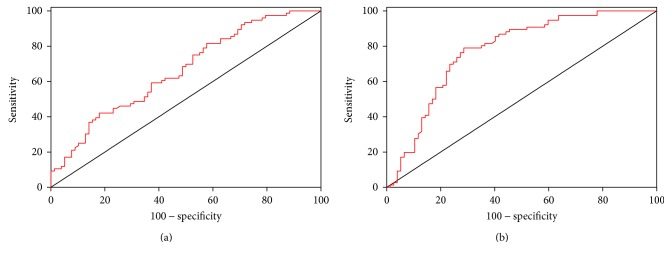
ROC curves of lactonase (a) and Lp-PLA_2_ (b) activities for the discrimination between controls and ASD (*n* = 156).

**Figure 5:**
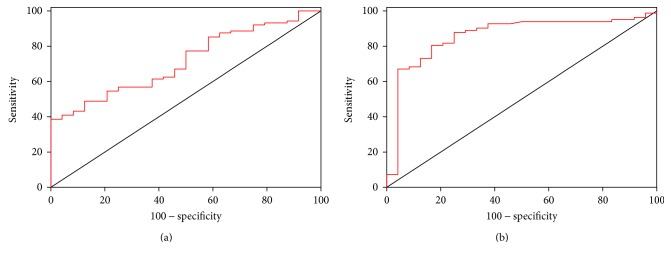
ROC curves of lactonase (a) and Lp-PLA_2_ (b) activity for the discrimination between ASD and RTT (only females, *n* = 121).

**Figure 6:**
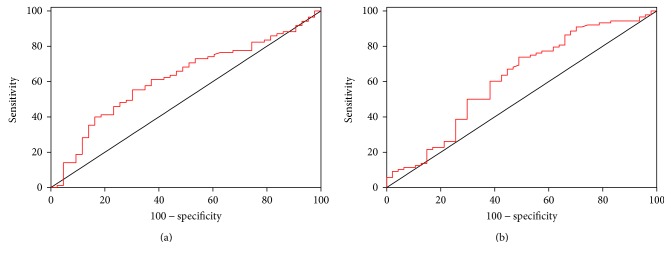
ROC curves of lactonase (a) and Lp-PLA_2_ (b) activity for the discrimination between controls and RTT (only females, *n* = 133).

The results presented suggest a possible role of lactonase and Lp-PLA_2_ as diagnostic biomarkers to discriminate ASD from controls and RTT. To address this hypothesis, a ROC analysis was performed for (1) controls versus ASD (considering both males and females) and (2) RTT versus ASD (considering only females). As displayed in Figures 4 and 5 (results summarized in Table 2), Lp-PLA_2_ appeared to be the most efficient parameter to discriminate between ASD patients and control subjects, and, in particular, girls with RTT from those with ASD (both sensitivity and specificity around 80%). On the contrary, lactonase appeared to have a lesser diagnostic potential (poor sensitivity) to discriminate between ASD and controls with respect to Lp-PLA_2_. Similarly, as suggested by previous analyses, neither lactonase nor Lp-PLA_2_ showed an acceptable accuracy in distinguishing controls from RTT patients (the best sensitivity and specificity around 60%, considering only female subsample) (Figure 6).

## 4. Discussion

One of the key concept in the definition of an ideal biomarker is that it should be “noninvasive, easily translatable to routine clinical testing, or eventually microfluidic high-throughput population screening and expedient serial monitoring” [34]. Factors that determine the clinical usefulness of a biomarker include the ease and cost of assessment, its performance characteristics (e.g., sensitivity, specificity, etc.), and the ability to reflect disease pathophysiology and to be helpful in the understanding the pathological process [35–37]. In light of these premises, our attempt to address the request of biological markers for the diagnosis of two “biomarker-orphan” diseases such as ASD and RTT was focused on PON-1 and Lp-PLA_2_. Indeed, these enzymes, besides fulfilling the requirements regarding cost and sample accessibility, might be implicated in the complex picture of immune dysregulation, inflammation, redox imbalance, and OxS characterizing both neurodevelopmental diseases.

The main finding of the present study was the high diagnostic potential revealed for PON-1-related lactonase and, mostly, Lp-PLA_2_ activities in discriminating ASD from both controls and RTT, reaching levels of specificity/sensitivity nearly the critical threshold of 80%. The possibility to distinguish between the two diseases is clinically relevant during the typical regression stage of RTT, when girls display many autistic features, such as loss of communication and social skills, poor eye contact, and lack of interest.

Another finding that deserves to be stressed is the different behavior of lactonase compared to the other two activities of PON-1, arylesterase, and paraoxonase. The substrates of the so-called promiscuous esterase activities are metabolites of handmade toxic chemicals such as organophosphorus compounds including insecticides and nerve agents [14, 38]. Our data suggest that neither RTT nor ASD is associated with the altered PON-1 activity for pollutants. The widely proposed etiological link between the exposure to environmental toxicants (pesticides, chemicals, phthalates, polychlorinated biphenyls, solvents, heavy metals, etc.) and the development of ASD [39] inspired the rationale of several studies on this topic. Most of these works have been focused on the association between single polymorphisms (SNPs), in primis, the SNP in position 192 (Q192R) of PON-1 affecting its activities and, thus, the ability to efficiently eliminate environmental toxicants [28, 40]. As recently reviewed by Rossignol et al. [41], the genetic studies only provide mixed support for an association between genetic polymorphisms in the PON-1 gene and ASD. Relevant in this context, D'Amelio et al. found that the Q192R polymorphism, which seems to be the strongest determinant of paraoxonase and lactonase activities (arylesterase is slightly influenced) [42, 43], was associated with a significantly increased risk of autism in American families living in North America but not in Italian families living in Italy [29]. The authors of this study suggested that the observed differences could be related to the level of exposure to pollutants and, thus, to the still unraveled interactions between genetic susceptibilities, toxicant exposures, and ASD risk [29].

Considering the sole arylesterase activity, the potential effects of environmental factors along with differences in the population studied (e.g., our subjects are older than those analyzed in other studies) and sample size can explain the discrepancy between our results (unvaried levels) and the ones from other laboratories (lower levels in ASD). On the other hand, the novel finding of the significant differences in lactonase activity we found may, at least in part, be due to differences in frequencies of the Q and R isoforms of Q192R polymorphism, associated with high and low catalytic efficiency, respectively, in the study groups. Unfortunately, the lack of the assessment of the frequency distribution of this SNP prevents us to address this hypothesis.

There is now a certain convergence that the third known activity of PON, lactonase, is the “primordial” of the enzyme and that endogen lipo-lactones are the physiological substrates [14, 16]. Despite the still unclear molecular mechanisms, it is increasing the idea that PON-1-lactonase contributes to the atheroprotective function of HDL by counteracting lipid peroxidation on LDL, HDL, and immune or nonimmune cells in a variety of diseases with an inflammatory component [12, 13, 36, 44]. The observed lactonase activity decrease in ASD could be interpreted as a weaker defense against lipoperoxidation damage, which have been repeatedly observed in both the brain and periphery of autistic patients [5, 40, 45, 46]. The altered redox state may represent the downstream phenomenon leading to the decrease in lactonase activity. Indeed, “in vitro” evidence suggests that oxidation, as well as glycation, may affect the ability of PON-1 to act as antioxidant and anti-inflammatory agent [47, 48], although it is still unclear to which extent these modifications impact on such proprieties.

Differently from PON-1, the “in vivo” action of Lp-PLA_2_ is suggested to be beneficial only in some physiological and pathological settings [44, 45], whereas in others (e.g., atherosclerosis) seems to be detrimental [18, 44, 45]. As described in some exhaustive reviews, the duality of this lipoprotein-associated should stem from multiple factors, including the following: (1) there is a wide spectrum of oxidized PLP expressing either pro- or anti-inflammatory activities; (2) equally, byproducts of enzyme catalysis are either pro- and anti-inflammatory; and (3) the direction of the activity is influenced by the lipoprotein carrier (LDL-associated Lp-PLA_2_ = proatherogenic; HDL-associated Lp-PLA_2_ = antiatherogenic).

Regardless of the aforementioned controversies and still open debate about both PON-1 and Lp-PLA_2_ biological functions, our novel observations suggest that in ASD but not in RTT, the decrease in these enzymes might contribute to the exacerbated lipid peroxidation processes underlying the disease. It is tempting to hypothesize that in the highly heterogeneous and complex ASD, OxS could likely arise from the cumulative influence, in prenatal and postnatal phase periods of environmental prooxidant factors, in particular heavy metals, air pollutants, toxins exposure, which most likely could adversely interact with still undefined genetic predisposition [45, 49, 50]. In turn, a “flaw” in one of the first physical defensive line results in higher vulnerability to these environmental toxicants and exacerbation of their adverse effects during these critical neurodevelopment periods. Regarding RTT, the synergy between environment-genetic background seems to play a minor role in OxS generation, and thus systemic defensive machineries, such as Lp-PLA_2_ and PON-1, could be less critical. The increase in reactive oxygen species (ROS) in RTT appears to reflect the deleterious vicious cycle characterized by upregulation of membrane-bound nicotinamide adenine dinucleotide phosphate oxidases (NOXs) and dysfunctional mitochondria (widely documented also in ASD), and an inadequate defensive enzymes responses [36]. The contribution of MECP2 deficiency in this aberrant process might stem from its ability to control, directly or indirectly, the expression of several redox-related genes [2, 11].

This novel study has anyway some limitations represented by the relatively small sample size, especially of female fraction in control and ASD groups affecting most likely the reliability and clinical significance of the observed associations. In addition, the cross-sectional design of this study did not allow us to definitely establish whether the examined lipoprotein-associated activities are merely novel biomarkers or a causal mediator of ASD. We are also aware that other biases or confounding factors not considered in this study (e.g., HDL-cholesterol concentration) might limit the reliability of our findings.

In spite of the aforementioned limitations, we believe that our study may provide a conceptual basis for larger investigations on this challenging topic. In particular, the demonstrated potential of lp-PLA_2_ to discriminate with good accuracy girls with ASD from controls and from those affected by RTT has a promising clinical application. In our opinion, children between 1 and 2 years of age could be the most appropriate targeted population for testing its diagnostic usefulness, for two main reasons: (1) ASDs are rarely diagnosed in children younger than 2 years of age because diagnosis is based on behavioral tests designed for older children [51] and (2) this age interval corresponds to the time window for autistic regression in RTT patients. More difficult is to hypothesize the use of lactonase for these purposes, due to the well-documented high inter- and intraindividual variability of PON-1 activities around that age [42, 52].

## 5. Conclusion

To the best of our knowledge, this is the first study to examine serum PON-1 lactonase and Lp-PLA_2_ activities in patients affected by ASD or RTT. The gathered evidence of a significant association between low levels of lactonase and, mostly, Lp-PLA_2_ activities in combination with ROC curve analysis outcomes suggests a potential use of these serum parameters as biomarkers in the diagnosis of ASD. Further studies on larger population sample are required to our findings.

## Figures and Tables

**Table 1:** Mean age and gender prevalence across the sample groups.

	Controls (*n* = 79)	RTT (*n* = 95)	ASD (*n* = 77)	Statistics
Gender F/M (*n*)	38/41	95/0	26/51	Controls versus RTT, *p* < 0.001 RTT versus ASD, *p* < 0.001
Age (years)	12 ± 7	16 ± 9	13 ± 8	Controls versus RTT, *p* = 0.055 RTT versus ASD, *p* = 0.940

Age is expressed as mean ± standard deviation (min–max). Difference between groups were evaluated by *χ*^2^ test (for gender) and Sidak post hoc test (for age).

**Table 2:** Performance of lactonase and Lp-PLA_2_ to discriminate between controls and ASD (*n* = 156) and between ASD and RTT (*n* = 121, only females).

Controls versus ASD						ASD versus RTT				
	AUC (95% CI)	*p* value	Cutoff	Sensitivity	Specificity	AUC (95% CI)	*p* value	Cutoff	Sensitivity	Specificity
Lp-PLA_2_	0.780 (0.705–0.854)	<0.001	11.2	70	77	0.858 (0.769–0.940)	<0.001	12.0	80	83
Lactonase	0.660 (0.576–0.746)	<0.001	96.4	59	63	0.714 (0.612–0.816)	<0.001	102	57	71

Cutoff points corresponding to the best combination between specificity and sensitivity.

## References

[1] Christodoulou J., Grimm A., Maher T., Bennetts B. (2003). RettBASE: the IRSA MECP2 variation database-a new mutation database in evolution. *Human Mutation*.

[2] Pecorelli A., Cervellati C., Hayek J., Valacchi G. (2016). OxInflammation in Rett syndrome. *The International Journal of Biochemistry & Cell Biology*.

[3] Armstrong D. D. (1997). Review of Rett syndrome. *Journal of Neuropathology and Experimental Neurology*.

[4] Baxter A. J., Brugha T. S., Erskine H. E., Scheurer R. W., Vos T., Scott J. G. (2015). The epidemiology and global burden of autism spectrum disorders. *Psychological Medicine*.

[5] Herbert M. R. (2010). Contributions of the environment and environmentally vulnerable physiology to autism spectrum disorders. *Current Opinion in Neurology*.

[6] Percy A. K. (2011). Rett syndrome: exploring the autism link. *Archives of Neurology*.

[7] Pecorelli A., Cervellati F., Belmonte G. (2016). Cytokines profile and peripheral blood mononuclear cells morphology in Rett and autistic patients. *Cytokine*.

[8] Leoncini S., De Felice C., Signorini C. (2015). Cytokine dysregulation in MECP2- and CDKL5-related Rett syndrome: relationships with aberrant redox homeostasis, inflammation, and ω-3 PUFAs. *Oxidative Medicine and Cellular Longevity*.

[9] Melnyk S., Fuchs G. J., Schulz E. (2012). Metabolic imbalance associated with methylation dysregulation and oxidative damage in children with autism. *Journal of Autism and Developmental Disorders*.

[10] James S. J., Cutler P., Melnyk S. (2004). Metabolic biomarkers of increased oxidative stress and impaired methylation capacity in children with autism. *The American Journal of Clinical Nutrition*.

[11] Cervellati C., Sticozzi C., Romani A. (2015). Impaired enzymatic defensive activity, mitochondrial dysfunction and proteasome activation are involved in RTT cell oxidative damage. *Biochimica et Biophysica Acta (BBA) - Molecular Basis of Disease*.

[12] Castellazzi M., Trentini A., Romani A. (2016). Decreased arylesterase activity of paraoxonase-1 (PON-1) might be a common denominator of neuroinflammatory and neurodegenerative diseases. *The International Journal of Biochemistry & Cell Biology*.

[13] Camps J., Marsillach J., Joven J. (2009). The paraoxonases: role in human diseases and methodological difficulties in measurement. *Critical Reviews in Clinical Laboratory Sciences*.

[14] Mackness M., Mackness B. (2015). Human paraoxonase-1 (PON1): gene structure and expression, promiscuous activities and multiple physiological roles. *Gene*.

[15] Harel M., Aharoni A., Gaidukov L. (2004). Structure and evolution of the serum paraoxonase family of detoxifying and anti-atherosclerotic enzymes. *Nature Structural & Molecular Biology*.

[16] Draganov D. I., Teiber J. F., Speelman A., Osawa Y., Sunahara R., La Du B. N. (2005). Human paraoxonases (PON1, PON2, and PON3) are lactonases with overlapping and distinct substrate specificities. *Journal of Lipid Research*.

[17] Rosenson R. S., Stafforini D. M. (2012). Modulation of oxidative stress, inflammation, and atherosclerosis by lipoprotein-associated phospholipase A2. *Journal of Lipid Research*.

[18] Rosenson R. S., Vracar-Grabar M., Helenowski I. (2008). Lipoprotein associated phospholipase A2 inhibition reduces generation of oxidized fatty acids: Lp-LPA2 reduces oxidized fatty acids. *Cardiovascular Drugs and Therapy*.

[19] Li D., Zhao L., Yu J. (2017). Lipoprotein-associated phospholipase a2 in coronary heart disease: review and meta-analysis.

[20] Mannheim D., Herrmann J., Versari D. (2008). Enhanced expression of Lp-PLA2 and lysophosphatidylcholine in symptomatic carotid atherosclerotic plaques. *Stroke*.

[21] Theilmeier G., De Geest B., Van Veldhoven P. P. (2000). HDL-associated PAF-AH reduces endothelial adhesiveness in apoE−/− mice. *The FASEB Journal*.

[22] Morgan E. N., Boyle E. M., Yun W. (1999). Platelet-activating factor acetylhydrolase prevents myocardial ischemia-reperfusion injury. *Circulation*.

[23] Cervellati C., Trentini A., Romani A. (2015). Serum paraoxonase and arylesterase activities of paraoxonase-1 (PON-1), mild cognitive impairment, and 2-year conversion to dementia: a pilot study. *Journal of Neurochemistry*.

[24] Cervellati C., Romani A., Bergamini C. M. (2015). PON-1 and ferroxidase activities in older patients with mild cognitive impairment, late onset Alzheimer’s disease or vascular dementia. *Clinical Chemistry and Laboratory Medicine*.

[25] Barathi S., Angayarkanni N., Pasupathi A. (2010). Homocysteinethiolactone and paraoxonase: novel markers of diabetic retinopathy. *Diabetes Care*.

[26] Thompson A., Gao P., Orfei L. (2010). Lipoprotein-associated phospholipase A2 and risk of coronary disease, stroke, and mortality: collaborative analysis of 32 prospective studies. *Lance*.

[27] Kasprzak M., Iskra M., Majewski W., Wielkoszyński T. (2009). Arylesterase and paraoxonase activity of paraoxonase (PON1) affected by ischemia in the plasma of patients with arterial occlusion of the lower limbs. *Clinical Biochemistry*.

[28] Paşca S. P., Nemeş B., Vlase L. (2006). High levels of homocysteine and low serum paraoxonase 1 arylesterase activity in children with autism. *Life Sciences*.

[29] D’Amelio M., Ricci I., Sacco R. (2005). Paraoxonase gene variants are associated with autism in North America, but not in Italy: possible regional specificity in gene-environment interactions. *Molecular Psychiatry*.

[30] Gaita L., Manzi B., Sacco R. (2010). Decreased serum arylesterase activity in autism spectrum disorders. *Psychiatry Research*.

[31] Paşca S. P., Dronca E., Nemeş B. (2010). Paraoxonase 1 activities and polymorphisms in autism spectrum disorders. *Journal of Cellular and Molecular Medicine*.

[32] Neul J. L., Kaufmann W. E., Glaze D. G. (2010). Rett syndrome: revised diagnostic criteria and nomenclature. *Annals of Neurology*.

[33] La Du B. N., Eckerson H. W. (1984). The polymorphic paraoxonase/arylesterase isozymes of human serum. *Federation Proceedings*.

[34] Rembach A., Stingo F. C., Peterson C. (2015). Bayesian graphical network analyses reveal complex biological interactions specific to Alzheimer’s disease. *Journal of Alzheimer's Disease*.

[35] Kadiiska M. B., Gladen B. C., Baird D. D. (2005). Biomarkers of oxidative stress study II: are oxidation products of lipids, proteins, and DNA markers of CCl4 poisoning?. *Free Radical Biology & Medicine*.

[36] Cervellati C., Wood P. L., Romani A. (2016). Oxidative challenge in Alzheimer’s disease: state of knowledge and future needs. *Journal of Investigative Medicine*.

[37] Dalle-Donne I., Rossi R., Colombo R., Giustarini D., Milzani A. (2006). Biomarkers of oxidative damage in human disease. *Clinical Chemistry*.

[38] Furlong C. E., Marsillach J., Jarvik G. P., Costa L. G. (2016). Paraoxonases-1, -2 and -3: what are their functions?. *Chemico-Biological Interactions*.

[39] Ye B. S., Leung A. O. W., Wong M. H. (2017). The association of environmental toxicants and autism spectrum disorders in children. *Environmental Pollution*.

[40] Rossignol D. A., Frye R. E. (2014). Evidence linking oxidative stress, mitochondrial dysfunction, and inflammation in the brain of individuals with autism. *Frontiers in Physiology*.

[41] Rossignol D. A., Genuis S. J., Frye R. E. (2014). Environmental toxicants and autism spectrum disorders: a systematic review. *Translational Psychiatry*.

[42] Costa L. G., Vitalone A., Cole T. B., Furlong C. E. (2005). Modulation of paraoxonase (PON1) activity. *Biochemical Pharmacology*.

[43] Huen K., Richter R., Furlong C., Eskenazi B., Holland N. (2009). Validation of PON1 enzyme activity assays for longitudinal studies. *Clinica Chimica Acta*.

[44] Martinelli N., Girelli D., Olivieri O. (2009). Novel serum paraoxonase activity assays are associated with coronary artery disease. *Clinical Chemistry and Laboratory Medicine*.

[45] Chauhan A., Chauhan V. (2006). Oxidative stress in autism. *Pathophysiology*.

[46] Yao Y., Walsh W. J., WR M. G., Praticò D. (2006). Altered vascular phenotype in autism: correlation with oxidative stress. *Archives of Neurology*.

[47] Ferretti G., Bacchetti T., Nègre-Salvayre A., Salvayre R., Dousset N., Curatola G. (2006). Structural modifications of HDL and functional consequences. *Atherosclerosis*.

[48] Aviram M., Rosenblat M., Billecke S. (1999). Human serum paraoxonase (PON 1) is inactivated by oxidized low density lipoprotein and preserved by antioxidants. *Free Radical Biology & Medicine*.

[49] Valenti D., de Bari L., De Filippis B., Henrion-Caude A., Vacca R. A. (2014). Mitochondrial dysfunction as a central actor in intellectual disability-related diseases: an overview of down syndrome, autism, fragile X and Rett syndrome. *Neuroscience & Biobehavioral Reviews*.

[50] McGinnis W. R. (2007). Could oxidative stress from psychosocial stress affect neurodevelopment in autism?. *Journal of Autism and Developmental Disorders*.

[51] Frustaci A., Neri M., Cesario A. (2012). Oxidative stress-related biomarkers in autism: systematic review and meta-analyses.

[52] Cole T. B., Jampsa R. L., Walter B. J. (2003). Expression of human paraoxonase (PON1) during development. *Pharmacogenetics*.

